# Satisfactory outcomes of post-operative subtalar extra-articular arthroereisis in juvenile flexible flat foot

**DOI:** 10.15537/smj.2023.44.1.20220607

**Published:** 2023-01

**Authors:** Abdulmonem M. Alsiddiky, Abdulaziz A. Alsubaie, Abdulaziz O. Almuhanna, Nawaf M. Alsubaie, Faisal A. Alsaleh, Hassan M. Alhazzani, Bader H. Alruwaily, Mohammad S. Alzahrani, Khalid A. Bakarman, Naief S. Alghnimei

**Affiliations:** *From the Department of Orthopedic Surgery, Research chair of spinal deformities (Alsiddiky, A. Alsubaie, Bakarman, Alghnimei), King Saud University, and from the College of Medicine (Almuhanna, M. Alsubaie, Alsaleh, Alhazzani, Alruwaily, Alzahrani), King Saud University, Riyadh, Kingdm of Saudi Arabia.*

**Keywords:** arthroereisis, flatfoot, sinus tarsi implant, patient satisfaction, surveys and questionnaires

## Abstract

**Objectives:**

To analyze the surgical outcomes of subtalar extra-articular arthroereisis and the patient/parent satisfaction regarding the foot’s shape, foot pain, ability to walk, ability to jump, and shoe wear.

**Methods:**

Our retrospective cross-sectional study was carried out through an online-based questionnaire to assess patient satisfaction postoperatively at 3 hospitals (King Khalid University Hospital, Sultan bin Abdulaziz Humanitarian City, and Dallah Hospital, Riyadh, Saudi Arabia) between the years 2014-2021.

**Results:**

A total of 65 patients participated in our study. Approximately 86.1% of them had the operation bilaterally. The most important aspects where patients noticed the most improvement were the foot’s shape (90.8%), pain (81.5%), and ability to walk (76.9%).

**Conclusion:**

Several studies have been carried out highlighting the surgical technique and complications of the procedure. However, a limited number of studies have been carried out to assess patient satisfaction with the procedure, especially in Saudi Arabia, as the procedure is considered relatively new in the region with insufficient data regarding it. Therefore, this study is considered one of the few articles regarding subtalar extra-articular arthroereisis in the region.

**F**lexible flatfoot (FFF) is a common malformation that can affect both children and adults. It is distinguished by a diminished or collapsed arch, excessive heel eversion while bearing weight, and forefoot abduction brought on by subtalar joint eversion.^1^

The majority of patients with flatfoot are asymptomatic, and it occasionally resolves on its own in certain cases. Additionally, tightness in the gastrocnemius or Achilles tendon, peroneus spasms, and medial column instability are frequently noted. Flexible flatfoot treatments in children remain debatable.^2^ Orthotics and conservative management are first-line treatments of FFF, but are sometimes inadequate; hence, surgical intervention is recommended for individuals who continue to complain on discomfort, loss of function, and easy fatiguability, or those who have deformity progression despite receiving conservative care.^3-6^ Several surgical procedures for flatfoot include tendon lengthening and transfer, osteotomies, subtalar arthroereisis, and arthrodesis.^7^ The subtalar arthroereisis procedure is a valid treatment option that refers to placing an artificial implant (these can include multiple types of implants including Kalix or Maxwell-Brancheau [MBA] implants) in the sinus tarsi to restrict excessive eversion of the subtalar joint and is often carried out in conjunction with Achilles tendon lengthening or gastrocnemius recession.^8,9^ To the best of our knowledge, the effects of subtalar arthroereisis on the flatfoot were not reported sufficiently due to the limited studies published regarding this treatment option in Saudi Arabia. Therefore, the purpose of this study is to evaluate the outcomes of treatment on postoperative pain, function, satisfaction, and shoe wear in our population in Riyadh, Saudi Arabia.

## Methods

We carried out a retrospective cross-sectional study of patients with FFF treated by subtalar extra-articular arthroereisis with Achilles tendon lengthening or gastrocnemius recession. All surgeries were carried out by our senior consultant pediatric orthopedic surgeon or under his supervision in 3 hospitals: King Khalid University Hospital, Sultan bin Abdulaziz Humanitarian City, and Dallah Hospital, Riyadh, Saudi Arabia, between 2014-2021. Consecutive sampling was used to collect our data.

The study included 65 juveniles with 121 symptomatic FFF (59 left feet and 62 right feet). There were 31 males and 34 females, with a mean age of 12.03 (range: 4-18) years. Among them, 56 patients were treated with bilateral flatfoot operations (86.1%). The mean operation time was 40.3 minutes per case (range: 25-50 minutes), and the mean perioperative blood loss was 5.2 mL per case (range: 2-24 mL). All 65 juveniles were followed up for a mean of 33.6 months (range: 4-89).

All patients diagnosed with FFF who failed conservative management and were between ages 4-18 years at the time of surgery, were included. All patients with rigid flatfoot or with prior surgical intervention were excluded. Verbal consents were obtained from all involved subjects or their guardians.

This study was approved by King Saud University Institutional Review Board (approval no.: E-22-6905).

**Figure 1:**
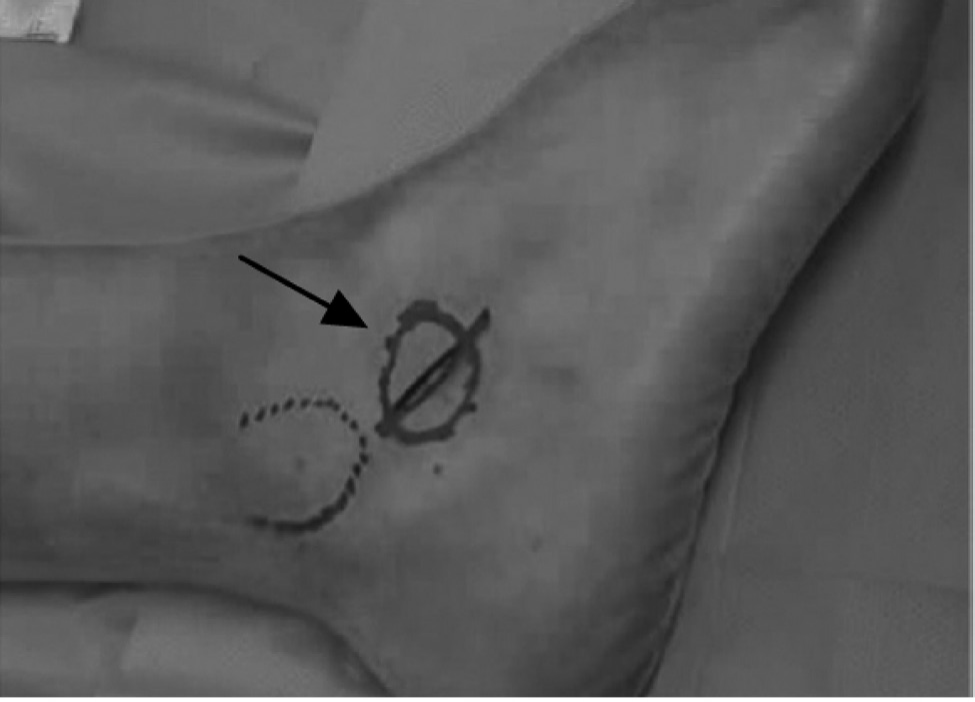
- The minimally invasive skin incision at the level of the sinus tarsi is approximately 2 cm.

We inserted a special implant (Kalix or MBA) into the tarsal canal that provides stability to the subtalar joint and prevents eversion. All procedures were carried out under general anesthesia as in-patient operations. All patients were in supine position with the ipsilateral hip lifted and draping was carried out. The first step to fulfill the procedure includes Achilles tendon lengthening or gastrocnemius muscle recession before the arthroreisis, then a 1-2 cm oblique skin incision is made over the sinus tarsi (Figure 1), and blunt subcutaneous dissection was carried out to expose the sinus tarsi. Then, a guide pin is inserted through the sinus tarsi and exits through a small incision medially. Trial implants were inserted until the appropriate size is determined. We chose the smallest implant that corrected the deformity and remained stable during the range of motion and examined with fluoroscopy. The skin was closed in layers, and a sterile dressing with a below-knee walking cast was applied. Postoperative measures include supporting devices such as a walking cast or brace to assist in walking for 6 weeks, and the patient was allowed for full weight bearing on the affected foot as tolerated.

Based on the online-based questionnaire administrated during the clinical interview, clinical variables, such as age, gender, pain, recurrence, satisfaction, complications, functional range of motion, and shoe wear, were analyzed. Results were categorized into significant, partial, or no improvement according to the patients’ impression when comparing the preoperative state with the postoperative outcome.

**Figure 2:**
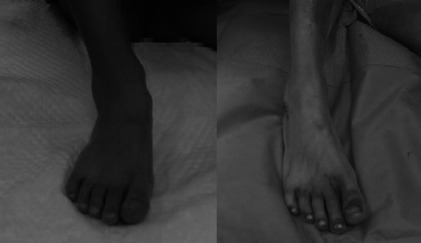
- Preoperative (left) and postoperative (right) anterior view showing realignment and correction of flatfoot deformity.

Figure 2 illustrates clinical photographs obtained before and after the subtalar arthroereisis procedure in a randomly selected patient.

**Figure 3:**
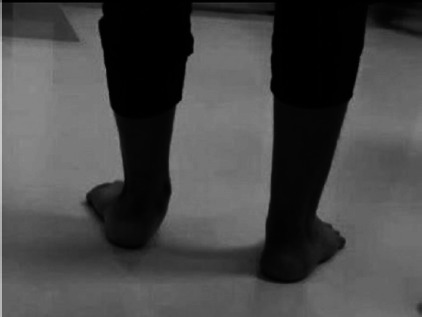
- Comparison of right foot which shows correction of hindfoot eversion and collapse of the arch postoperatively, in comparison to the left foot prior to operation.

Figure 3 exhibits a clinical photograph obtained during a follow-up visit between 2 scheduled operations for the correction of each foot in a randomly selected patient.

### Statistical analysis

Data were analyzed using the Statistical Package for the Social Sciences, version 22.0 (IBM Corp., Armonk, NY, USA). Continuous variables were expressed as mean ± standard deviation (SD), and categorical variables were expressed as percentages. The Chi-squared test was used for categorical variables. A *p*-value of <0.05 was considered significant.

## Results

The study included 65 patients in all, with a mean age of 12.03±3.75 years. Data are shown in Table 1. The majority (86.1%) of patients were operated on bilaterally (right and left), and the vast majority (90.8%) have noted postoperative improvement in the shape of the foot, whether significant (60%) or partial (30.8%). In terms of pain, 81.5% have reported an improvement in foot pain post-surgery (41.5% significant and 40% partial improvement). Approximately 76.9% reported improvement in the ability to walk long or short distances. The capacity to jump and play significantly improved in 35.4% of patients postoperatively, whereas 35.4% were unable to do so. More than half (55.4%) of patients stated that being able to wear shoes postoperatively had significantly improved. Only 4 (6.1%) had the metal implant removed after surgery, and 2 of them did so within the first 3 years. When data were analyzed according to the patient’s age group (Table 2), we found no significant differences in any of the studied characteristics (*p*>0.05). However, significant clinical improvement was higher in the younger age group (<12 years) compared to those aged ≥12 years in foot shape (61.5% vs. 59.0%), foot pain (46.1% vs. 38.5%), ability to jump and play (46.1% vs. 28.2%), and ability to wear shoes (57.7% vs. 53.8%).

**Table 1:** - Characteristics of the patients (N=65).

Variables	n (%)
Age, mean	12.03
***On which foot was the operation carried out?***	
Right Left Both feet	6 (9.2) 3 (4.6) 56 (86.1)
***How much improvement was recognized in the shape of the foot, comparing before and after the operation?***	
Significant improvement Partial improvement No improvement	39 (60.0) 20 (30.8) 6 (9.2)
***How much improvement was noticed in terms of foot pain, comparing before and after the operation?***	
Significant improvement Partial improvement No change in painIncrease in pain	27 (41.5) 26 (40.0) 6 (9.2) 6 (9.2)
***How much improvement was noticed in the ability to walk, comparing before and after the operation?***	
Ability to walk long distances Ability to walk short distances Unable to walk	27 (41.5) 23 (35.4) 15 (23.1)
***How much improvement was noticed in the ability to jump and play, comparing before and after the operation?***	
Significant improvement Partial improvement Unable to jump and play	23 (35.4) 19 (29.2) 23 (35.4)
***How much improvement was noticed in the ability to wear shoes, comparing before and after the operation?***	
Significant improvement Partial improvement Unable to wear shoes	36 (55.4) 14 (21.5) 15 (23.1)
***Was the metal fragment extracted after the procedure? (any duration postoperative)***	
Yes No	4 (6.1) 61 (93.8)
***If the answer to the previous question was yes, which foot was the metal fragment removed from?***	
Right Left Both feet	1 (25.0) 2 (50.0) 1 (25.0)
***What was the duration before removal of the metal fragment?***	
<1 year 1-3 years >3 years	1 (25.0) 2 (50.0) 1 (25.0)

Values are presented as numbers and precentages (%).

**Table 2:** - Characteristics of the patients by age group.

Variables	<12 years (n=26)	≥12 years (n=39)	*P*-values
***On which foot was the operation carried out?***			
Right Left Both feet	3 (11.5) 0 (0.0) 23 (88.5)	3 (7.7) 3 (7.7) 33 (84.6)	0.32
***How much improvement was recognized in the shape of the foot, comparing before and after the operation?***			
Significant improvement Partial improvement No improvement	16 (61.5) 10 (38.5) 0 (0.0)	23 (59.0) 10 (25.6) 6 (15.4)	0.088
***How much improvement was noticed in terms of foot pain, comparing before and after the operation?***			
Significant improvement Partial improvement No change in painIncrease in pain	12 (46.1) 12 (46.1) 0 (0.0) 2 (7.7)	15 (38.5) 14 (35.9) 6 (15.4) 4 (10.3)	0.192
***How much improvement was noticed in the ability to walk, comparing before and after the operation?***			
Ability to walk long distances Ability to walk short distances Unable to walk	10 (38.5) 11 (42.3) 5 (19.2)	17 (43.6) 12 (30.8) 10 (25.6)	0.618
***How much improvement was noticed in the ability to jump and play, comparing before and after the operation?***			
Significant improvement Partial improvement Unable to jump and play	12 (46.1) 6 (23.1) 8 (30.8)	11 (28.2) 13 (33.3) 15 (38.5)	0.326
***How much improvement was noticed in the ability to wear shoes, comparing before and after the operation?***			
Significant improvement Partial improvement Unable to wear shoes	15 (57.7) 5 (19.2) 6 (23.1)	21 (53.8) 9 (23.1) 9 (23.1)	0.928
***Was the metal fragment extracted after the procedure? (any duration postoperative)***			
Yes No	2 (7.7) 24 (92.3)	2 (5.1) 37 (94.9)	0.673
***If the answer to the previous question was yes, which foot was the metal fragment removed from?***			
Right Left Both feet	0 (0.0) 1 (50.0) 1 (50.0)	1 (50.0) 1 (50.0) 0 (0.0)	0.368
***What was the duration before removal of the metal fragment?***			
<1 year 1-3 years >3 years	0 (0.0) 2 (100) 0 (0.0)	1 (50.0) 0 (0.0) 1 (50.0)	0.135

Values are presented as numbers and precentages (%).

**Table 3:** - Characteristics of the patients by foot.

Variables	One foot (right or left), n=9	Two feet (n=56)	*P*-values
***On which foot was the operation carried out?***			
Significant improvement Partial improvement No improvement	5 (55.6) 1 (11.1) 3 (33.3)	34 (60.7) 19 (33.9) 3 (5.4)	0.019*
***How much improvement was recognized in the shape of the foot, comparing before and after the operation?***			
Significant improvement Partial improvement No change in painIncrease in pain	4 (44.4) 4 (44.4) 1 (11.1) 0 (0.0)	23 (41.1) 22 (39.3) 5 (8.9) 6 (10.7)	0.783
***How much improvement was noticed in the ability to walk, comparing before and after the operation?***			
Ability to walk long distances Ability to walk long distances Unable to walk	2 (22.2) 5 (55.6) 2 (22.2)	25 (44.6) 18 (32.1) 13 (23.2)	0.343
***How much improvement was noticed in the ability to jump and play, comparing before and after the operation?***			
Significant improvement Partial improvement Unable to jump and play	1 (11.1) 3 (33.3) 5 (55.6)	22 (39.3) 16 (28.6) 18 (32.1)	0.223
***How much improvement was noticed in the ability to wear shoes, comparing before and after the operation?***			
Significant improvement Partial improvement Unable to wear shoes	4 (44.4) 1 (11.1) 4 (44.4)	32 (57.1) 13 (23.2) 11 (19.6)	0.244
***Was the metal fragment extracted after the procedure? (any duration postoperative)***			
Yes No	0 (0.0) 9 (100)	4 (7.1) 52 (92.9)	0.408
***If the answer to the previous question was yes, which foot was the metal fragment removed from?***			
Right Left Both feet	0 (0.0) 0 (0.0) 0 (0.0)	1 (25.0) 2 (50.0) 1 (25.0)	
***What was the duration before removal of the metal fragment?***			
<1 year 1-3 years >3 years	0 (0.0) 0 (0.0) 0 (0.0)	1 (25.0) 2 (50.0) 1 (25.0)	

Values are presented as numbers and precentages (%).

When characteristics were studied according to the operated foot, either unilateral or bilateral (Table 3), we found statistically significant results when 2 feet were operated on, showing that improvement in the shape of the foot was significantly (*p*=0.019) higher than those who were operated on one foot only, with significant improvement of (60.7% vs. 55.6%) and partial improvement of (33.9% vs. 11.1%). Other characteristics in Table 3 were insignificant.

## Discussion

Clinical assessment and findings of the distinctive radiographic anomalies are usually used to diagnose FFF.^1^ Despite the ease of making a diagnosis, therapeutic principles and surgical indications are still controversial.^2^ Some experts recommended that only functioning flatfeet should be treated.^3^ However, distinguishing between morphological and functional flatfoot is not always easy; more often than not, surgical therapy is needed when the patient complains of pain, discomfort, early weariness, and limits in routine activities.^4,5^ The largest impact of FFF on patients is the impact on quality of life, as when patients are symptomatic it prevents them from performing their daily activities, this is especially important in the pediatric age group, as walking, running, and playing are integral factors in childhood. Arthroereisis is one of the most commonly carried out procedures for the treatment of FFF. Therefore, this study aimed to assess and analyze the outcomes of post-subtalar arthroereisis procedure by improving pain, function, satisfaction, and shoe wear in Riyadh, Saudi Arabia. Results of the current study revealed a significant clinical improvement in foot shape, pain, ability to jump and play, ability to walk long distances, and ability to wear shoes. Our results are also in line with a previous similar study among juveniles showing subtalar joint arthroereisis can be beneficial in the treatment of FFF.^6^ When compared to operations such as midfoot or hindfoot osteotomies or arthrodesis, arthoereisis has reduced morbidity for patients peri-operatively. The risk of nonunion and immobilization could occur, and arthroereisis is considered a simple surgical procedure.^6^ Similarly, when considering arthroereisis, all authors reporting results on different cohorts (noncomparative studies) concluded that this minimally invasive procedure was an ‘optimal’ technique for the correction of FFF in children and adults, providing clinical and radiological satisfactory outcomes.^7-9^ A recently published review by Tan et al^10^ examined the usage of arthroereisis in pes planus and concluded that arthroereisis was efficient in alleviating symptoms and deformities, a finding in agreement with our study results. Previous studies also reported satisfactory results that were comparable to those of the current research. Following the surgery in an adult population, Voegeli et al^11^ discovered that 74% of patients were entirely happy or had only minor limits, such as discomfort or impairments in everyday life. Metcalfe et al^12^ observed patient satisfaction percentages ranging from 79-100% in pediatrics. Brancheau et al^13^ carried out a study using a patient questionnaire to assess MBA screw arthroereisis in adolescents and adults with symptomatic flexible flatfeet. This was a self-created questionnaire demonstrating that 95% of patients experienced alleviation from their concerns following therapy. Furthermore, 83% of patients reported no daily discomfort, and 62% participated in sports at a greater level.

In contrast to the current study findings which showed no statistically significant association between the procedure outcome and the patient’s age, Kubo et al^14^ found that age at the time of surgery appears to be a key factor affecting the outcome, with patients ages between 9-12 years considered the optimum. Younger individuals are at a higher risk of recurrence, whereas older patients have less effective results, most likely due to the foot’s limited reconstruction ability.^14^

### Study limitations

The number of patients in our study was limited due to the fact that only patients operated on by one senior consultant in the 3 hospitals were involved in our study. The follow-up duration also varied due to the same reasoning.

We recommend a multi-center study that assesses the satisfaction of patients and parents for subtalar extra-articular arthroereisis. In addition, we recommend a multi-center study that applies subjective measures such as radiographic imaging to assess postoperative outcomes.

In conclusion, FFF is a common condition with multiple treatment options, surgical management is a valid option, especially for cases where conservative management has failed. Subtalar extra-articular arthroereisis is one of the common procedures for the treatment of FFF, and it has shown promising results. Our results concluded that the procedure had satisfactory outcomes postoperatively specifically in regard to the shape of the foot and pain.
